# Analysis of *Globodera rostochiensis* effectors reveals conserved functions of SPRYSEC proteins in suppressing and eliciting plant immune responses

**DOI:** 10.3389/fpls.2015.00623

**Published:** 2015-08-11

**Authors:** Shawkat Ali, Maxime Magne, Shiyan Chen, Natasa Obradovic, Lubna Jamshaid, Xiaohong Wang, Guy Bélair, Peter Moffett

**Affiliations:** ^1^Département de Biologie, Université de SherbrookeSherbrooke, QC, Canada; ^2^Horticulture R & D Centre, Agriculture and Agri-Food CanadaSt-Jean-sur-Richelieu, QC, Canada; ^3^Division of Biological and Environmental Sciences and Engineering, Center for Desert Agriculture, King Abdullah University of Science and TechnologyThuwal, Saudi Arabia; ^4^School of Integrative Plant Science, Cornell UniversityIthaca, NY, USA; ^5^US Department of Agriculture, Robert W. Holley Center for Agriculture and Health, Agricultural Research ServiceIthaca, NY, USA

**Keywords:** *Globodera*, plant-parasitic nematode, cyst nematodes, NB-LRR proteins, effector proteins, PAMP-triggered immunity (PTI), SPRYSEC

## Abstract

Potato cyst nematodes (PCNs), including *Globodera rostochiensis* (Woll.), are important pests of potato. Plant parasitic nematodes produce multiple effector proteins, secreted from their stylets, to successfully infect their hosts. These include proteins delivered to the apoplast and to the host cytoplasm. A number of effectors from *G. rostochiensis* predicted to be delivered to the host cytoplasm have been identified, including several belonging to the secreted SPRY domain (SPRYSEC) family. SPRYSEC proteins are unique to members of the genus *Globodera* and have been implicated in both the induction and the repression of host defense responses. We have tested the properties of six different *G. rostochiensis* SPRYSEC proteins by expressing them in *Nicotiana benthamiana* and *N. tabacum*. We have found that all SPRYSEC proteins tested are able to suppress defense responses induced by NB-LRR proteins as well as cell death induced by elicitors, suggesting that defense repression is a common characteristic of members of this effector protein family. At the same time, *Gr*SPRYSEC-15 elicited a defense responses in *N. tabacum*, which was found to be resistant to a virus expressing *Gr*SPRYSEC-15. These results suggest that SPRYSEC proteins may possess characteristics that allow them to be recognized by the plant immune system.

## Introduction

Plants can be infected by multiple types of pathogens, including microbial and viral pathogens, as well as metazoan parasites such as insects and plant-parasitic nematodes. Plants mount defenses against pathogens through the recognition of pathogen-associated molecular patterns (PAMPs) in the extracellular space. This PAMP-triggered immunity (PTI) is often mediated by transmembrane receptor-like kinases and is often sufficient to confer resistance to non-adapted pathogens (Monaghan and Zipfel, 2012; Schwessinger and Ronald, 2012). In turn, host-adapted biotrophic pathogens produce proteins known as effectors either to the apoplast or to the host cell cytoplasm and many microbial effectors delivered to the cytoplasm have been shown to interfere with PTI mechanisms (Macho and Zipfel, 2015). Like microbial pathogens, nematodes, and insect parasites have also been shown to secrete effector proteins, some of which are thought to be delivered to the host cytoplasm where they inhibit PTI mechanisms (Goverse and Smant, 2014; Jaouannet et al., 2014). As a second line of defense, plants encode nucleotide-binding, and leucine-rich repeat (NB-LRR) proteins, which can recognize intracellularly-delivered effector proteins and induce effector-triggered immunity (ETI, Chisholm et al., 2006). ETI induces a much stronger response than PTI, often inducing a type of cell death known as the hypersensitive response (HR).

Potato cyst nematodes (PCNs), including *Globodera rostochiensis* and *G. pallida*, parasitize several *Solanaceous* plants, including potato, tomato, and eggplant and are a major impediment to potato production worldwide (Jones et al., 2013). Prior to parasitism, infective second-stage juveniles (J2) hatch from eggs in the soil and find their way to host plant roots by attraction to root diffusates. The J2 uses its stylet (a hollow protrusible mouth spear) to mechanically penetrate the root and migrate toward the root vasculature. Once at the vasculature the juvenile becomes a highly specialized obligate sedentary endoparasite by selecting a cell to establish a unique feeding structure called a syncytium (Davis et al., 2004). This process is thought to be mediated in large part by secretions from the stylet, including apoplastic effectors as well as effectors that are delivered to the cytoplasm of the infected cell. Nematode effectors are produced in the pharyngeal gland cells (two subventral and one dorsal) and delivered either to the apoplast or the host cell cytoplasm through the stylet (Mitchum et al., 2013). These include cell wall-modifying enzymes and small peptides secreted to the apoplast as well as a number of cytoplasmic effectors (Mitchum et al., 2012, 2013). The functions of the latter are largely unknown, although a number have recently been shown to inhibit plant defense responses, similar to microbial effectors (Goverse and Smant, 2014).

The secreted SPRY domain (SPRYSEC) family of effector proteins is specific to *Globodera* spp. and has undergone significant expansion in these species (Cotton et al., 2014). One member of this family, *Gp*-RBP-1, has been shown to be highly polymorphic and certain variants are recognized by the NB-LRR protein Gpa2 (Sacco et al., 2009). The latter report showed that Gpa2 recognizes *Gr*-RBP-1 inside the plant cell and from this it is assumed that SPRYSEC proteins are delivered to, and function in, the host cytoplasm. The *Gp-RBP-1* gene appears to be under selection in natural populations of *G. pallida*, as does the gene encoding the Gpa2 recognition co-factor RanGAP2 in *Solanum* species, suggesting an important co-evolutionary interaction (Carpentier et al., 2012, 2013). A *G. rostochiensis* SPRYSEC protein, *Gr*SPRYSEC-19 has been shown to interact with an NB-LRR protein and to inhibit plant defense responses (Rehman et al., 2009; Postma et al., 2012).

In this study we have characterized six different SPRYSEC proteins from *G. rostochiensis* by expressing them in different plants either by transient Agrobacterium-mediated expression (agroinfiltration) or from a viral vector based on *Potato virus X* (PVX). Using transient expression assays, we found that all six SPRYSEC proteins were able to suppress the cell death induced by two different elicitors as well as by two different NB-LRR proteins in *Nicotiana benthamiana* and/or *N. tabacum*. The same proteins were also able to inhibit death-independent anti-viral responses induced by two different NB-LRR proteins in *N. benthamiana*. These results suggest that SPRYSEC proteins are able to inhibit multiple aspects of the plant immune response and that this is likely a characteristic common to most SPRYSEC proteins. At the same time, we show that *Gr*SPRYSEC-15 confers resistance to a recombinant virus expressing the latter protein, suggesting that recognition of SPRYSEC proteins by the plant immune system may also be common for this class of proteins.

## Material and methods

### Bacterial strains, plants, and culture conditions

PVX and pEAQ-based expression vectors were delivered using *Agrobacterium tumefaciens* strain GV3101 or strain C58C1, respectively, by agroinfiltration as described (Ali et al., 2015). Plants were grown at 22°C, 50% humidity in a controlled growth chamber condition with 14/10 h light/dark cycle.

### Cloning of effectors and in planta expression assays

Cloning of SPRYSEC proteins was carried out as previously described (Ali et al., 2015). Briefly, *G. rostochiensis* pre-parasitic second-stage juveniles (pre-J2s) or *G. rostochiensis* infected potato roots from Quebec populations (Boucher et al., 2013) were used for RNA isolation using either Trizol or RNeasy Mini Kit (QIAGEN). cDNA was synthesized from mRNA by reverse transcription using an oligo dT primer and superscript III RT (cDNA Synthesis with SuperScript® III system, Invitrogen Life Technology). SPRYSEC genes were amplified with specific primers without their cognate signal peptide (SP) (Table S1) using high fidelity KOD hot start DNA polymerase (EMD Millipore).

PCR fragments were gel purified and cloned into pDONR207 entry clone by BP clonase (Invitrogen Life Technology) following the manufacturer's instructions and transformed into *E. coli* DH5α. Inserts in the resulting entry clones were sequenced and recombined into gateway compatible binary vector pEAQ35S, and PVX based vectors PVX and PVX-HB (Ali et al., 2015) by LR clonase reaction (Invitrogen Life Technology), following the manufacturer's instructions. The resulting pEAQ35S clones were then transformed into electro competent *A. tumefaciens* strain C58C1 and the PVX expression vectors were transformed into GV3101 strain containing the helper plasmid pJIC SA_Rep (Hellens et al., 2000). Four to six-week-old *N. tabacum, N. benthamiana*, potato, and tomato plants were agroinfiltrated as previously described (Ali et al., 2015).

### Cell death and disease resistance suppression assays

Cell death suppression experiments were carried out as previously described (Ali et al., 2015). Briefly, *Agrobacterium* strains carrying the SPRYSEC effectors either in the pEAQ35S or PVX constructs were resuspended in 10 mM MgCl_2_ such that all effector carrying strains were infiltrated at a final OD_600_ of 0.2 and the cell death inducers at a final OD_600_ of 0.1. The viral suppressor of RNA silencing of Turnip Crinkle Virus (TCV), P38 (Qu et al., 2003; Thomas et al., 2003) was also included at a final OD_600_ of 0.1. When assessing effectors for cell death suppression, a control with cell death inducer and empty vector was always infiltrated on the opposite side of the leaf. All constructs were agroinfiltrated on two different leaves of two different plants in both *N. benthamiana*, and *N. tabacum*, and all experiments were repeated at least three times. Cell death symptoms were scored 3–5 days-post-inoculation (DPI). Cell death inducers included INF1from *Phytophthora infestans*, a constitutively active version of the Rx protein (AtRx) (Kamoun et al., 1998; Bendahmane et al., 2002; Rairdan and Moffett, 2006), the Rx protein, together with its elicitor, the PVX coat protein (CP), the *P. sojae* elicitor PiNPP (Bendahmane et al., 2002; Kanneganti et al., 2006) and Bs2 plus AvrBs2 (Tai et al., 1999). Two cell death inducers Avh238 and Avh241 from *P. sojae* and a known cell death suppressor, Avr3a from *P. infestans*, were included as controls (Bos et al., 2010; Wang et al., 2011; Yu et al., 2012). Suppression of virus resistance induced by N and Rx was carried out as previously described (Rairdan et al., 2008; Bhattacharjee et al., 2009).

### Gene expression analysis by quantitative RT-PCR (qRT-PCR)

mRNA from different nematode life stages was extracted and used for first-strand cDNA synthesis as previously described (Chronis et al., 2013; Ali et al., 2015). Quantitative real-time RT-PCR (qRT-PCR) assays were used to determine the expression profile of five SPRYSEC encoding genes in *G. rostochiensis*. The qRT-PCR assay was carried out in a 25 μl reaction volume containing iQ SYBR Green Supermix (Bio-Rad Laboratories), 500 nM of both forward and reverse primers (Table S1) and 1 μl cDNA. mRNA samples for each developmental stage were prepared from two independent experiments and used for cDNA synthesis. All qPCR assays consisted of three technical replicates for each cDNA sample. qPCR was started at 98°C for 3 min, followed by 40 cycles of 98°C for 20 s and 60°C for 1 min, and then a final step of 72°C for 5 min. The *G. rostochiensis* β*-actin* gene (*Gract-1*) (EF437156) was used as an endogenous reference for data analysis using the 2^−ΔΔCt^ method (Lu et al., 2009). For each developmental stage, 2^−ΔΔCt^ represented the amount of the target gene expression that was normalized to *Gract-1* and relative to a calibrator that had the lowest expression in the cyst or other life stages.

## Results

### Identification and transcriptional profiling of *G. rostochiensis* SPRYSEC proteins

We have previously undertaken a survey of putative *G. rostochiensis* effector proteins based on published reports and *in silico* predictions using EST databases (Ali et al., 2015). The selection of candidates was based in part on the prediction of the presence of N-terminal signal peptide (SP) in the predicted proteins, which is required for secretion from eukaryotic pathogens before being delivered to the host cytoplasm or apoplast (Win et al., 2012). Our previous analysis identified 37 candidate effectors and we reported on the characterization of several putative apoplastic effectors (Ali et al., 2015). The latter study also identified six SPRYSEC proteins, which we characterize herein. These sequences match predicted *G. rostochiensis* SPRYSEC proteins previously reported (Postma et al., 2012) or present in GenBank (Table S2), including *Gr*SPRYSEC-4, *Gr*SPRYSEC-5, *Gr*SPRYSEC-8, *Gr*SPRYSEC-15, *Gr*SPRYSEC-18, and *Gr*SPRYSEC-19. Two SPRYSEC effector sequences differing from previously published sequences are listed in Table S2 and have been deposited in Genbank (accessions KF963513.1 and KF963514.1, respectively) and are shown in Figure S4. The version of SPRYSEC-15 described herein possesses a frame shift due to a nucleotide insertion and an early stop codon predicted to eliminate the last 28 amino acids of the protein compared to the reported sequence (Table S2, Figure S4). This difference was not due to PCR error as sequencing of four clones amplified from different cysts yielded identical sequences, indicating that the latter is a *bone fide* sequence variant.

Quantitative real-time RT-PCR (qRT-PCR) was used to determine the expression profile of five of the SPRYSEC-encoding genes through the five nematode developmental stages: egg, pre-J2 and parasitic second-, third-, and fourth-stage juveniles (par-J2, J3, and J4). The five genes encoding for members of the SPRYSEC family we tested all showed high levels of expression both in the pre-J2 and early parasitic stages up until 10 DPI (Figure S1).

### Transient *in planta* expression of *G. rostochiensis* SPRYSEC proteins

SPRYSEC proteins are homologous to *Gp*-RBP-1, which has been shown to be recognized inside the plant cell by Gpa2 (Sacco et al., 2009). It is therefore highly likely that SPRYSEC proteins function inside the plant cell. The six SPRYSEC encoding genes were thus cloned without their SP in three different Gateway compatible constructs: pEAQ35S; PVX, and PVX-HB (Ali et al., 2015). The pEAQ35S-SPRYSEC binary constructs allow for transient and localized expression at the infiltration site in plant leaves via agroinfiltration. PVX-derived vectors allow for localized and systemic expression of SPRYSEC effector proteins *in planta* from the genome of PVX. PVX-HB was used for the delivery of effectors into potato cultivars expressing the *Rx* gene, which confers resistance to PVX (Bendahmane et al., 1999). We expressed the six SPRYSEC proteins transiently and systemically in *N. benthamiana, N. tabacum*, tomato, and potato (Ali et al., 2015), both by agroinfiltration and agroinfection from pEAQ35S and the PVX vectors, respectively. Phenotypes were assessed either for the induction of visible changes in agroinfiltrated leaf patches or induction of visual morphological changes in systemically infected plants. Although none of the *Gr*SPRYSEC proteins induced any apparent effect in tomato or potato (data not shown), several effects were observed in *N. benthamiana*, and *N. tabacum* (see below).

### GrSPRYSEC-15 induces cell death in *N. tabacum* and cholorosis in *N. benthamiana*

**Figure 1:**
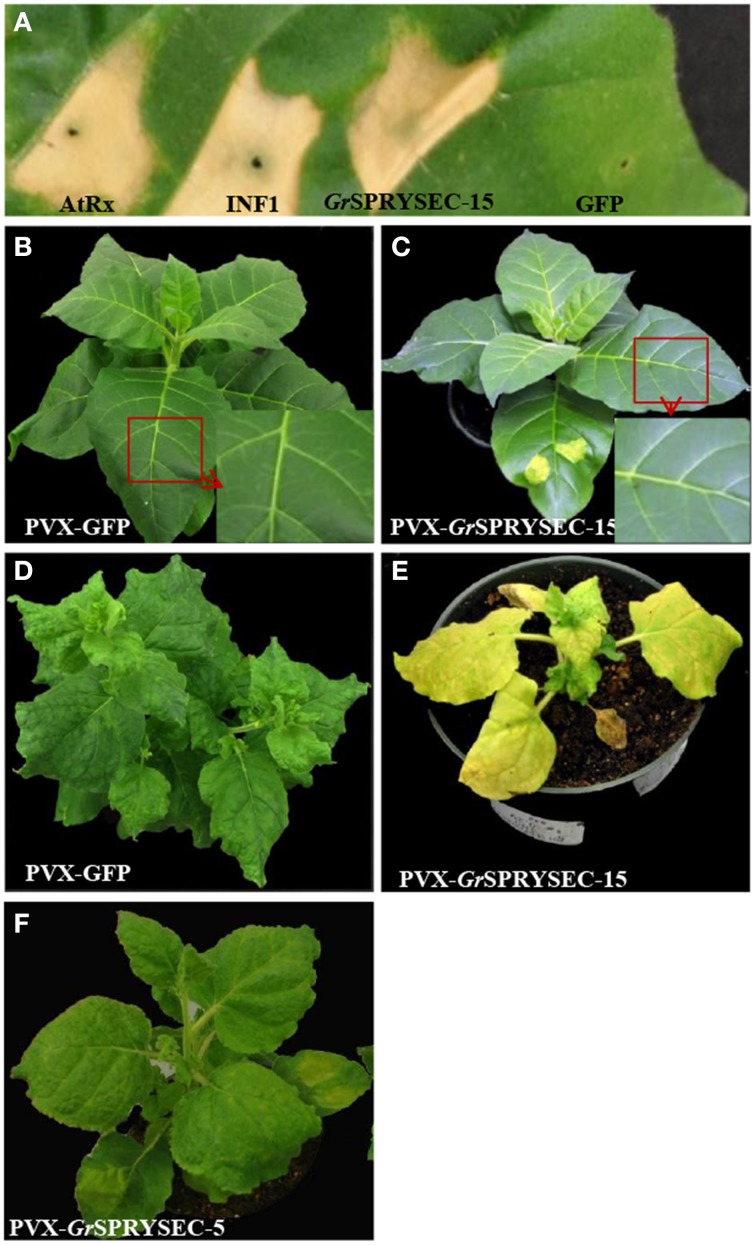
***Gr*SPRYSEC-15 induces a hypersensitive response in ***N***. ***tabacum*** and chlorosis in ***N. benthamiana***. (A)** *N. tabacum* leaves were infiltrated with *Agrobacterium* carrying pEAQ35S expressing AtRx, INF1, *Gr*SPRYSEC-15, or GFP as indicated. Leaves were photographed at 4 DPI. **(B)** *N. tabacum* inoculated by agroinfiltration with PVX-GFP vector or **(C)** PVX-*Gr*SPRYSEC-15. Pictures were taken at 14 DPI. Insets show close ups of leaves displaying typical PVX symptoms and the sites of agroinfiltration of PVX-*Gr*SPRYSEC-15 are seen as yellowish spots in **(C)**. **(D)** *N. benthamiana* inoculated with PVX-GFP vector or **(E)** PVX-*Gr*SPRYSEC-15 or **(F)** PVX-GrSPRYSEC-5. Plants were photographed at 21 DPI.

To investigate whether *G. rostochiensis* SPRYSEC proteins induce a localized effect when expressed in a defined leaf patch, we expressed the six SPRYSEC proteins in *N. benthamiana* and *N. tabacum*, using the binary construct pEAQ35S. INF1 from *P. infestans* and a constitutively active version of the Rx protein (AtRx), which is known to induce cell death in *N. benthamiana* and *N. tabacum*, were used as positive controls (Kamoun et al., 1998; Bendahmane et al., 2002; Rairdan and Moffett, 2006). Of the six SPRYSEC effectors, only expression of *Gr*SPRYSEC-15 presented a visible phenotype, inducing an HR in *N. tabacum*, showing the strength and timing similar to that induced by INF1 and AtRx (Figure 1A). Similarly, a strong HR was induced in the infiltrated area of *N. tabacum* leaves 3–4 days after agroinfiltration of PVX-*Gr*SPRYSEC-15 but not with PVX-GFP (Figures 1B,C). At the same time, whereas *N. tabacum* plants inoculated with PVX-GFP showed systemic viral symptoms, including light chlorosis around leaf veins, PVX- *Gr*SPRYSEC-15 showed no apparent systemic movement (Figures 1B,C). This result suggests that recognition of *Gr*SPRYSEC-15 by an endogenous disease resistance protein renders the recombinant PVX clone avirulent on *N. tabacum*.

In *N. benthamiana* the expression of *Gr*SPRYSEC-15 from pEAQ35S did not induce HR-like symptoms (data not shown) but its systemic expression did result in severely chlorotic and dwarfed plants compared to PVX-GFP infected plants (Figures 1D,E). No other SPRYSEC protein induced similar symptoms and a representative result (PVX-*Gr*SPRYSEC-5) is shown in Figure 1F.

### Suppression of immunity-associated cell death by *G. rostochiensis* SPRYSEC effectors

**Figure 2:**
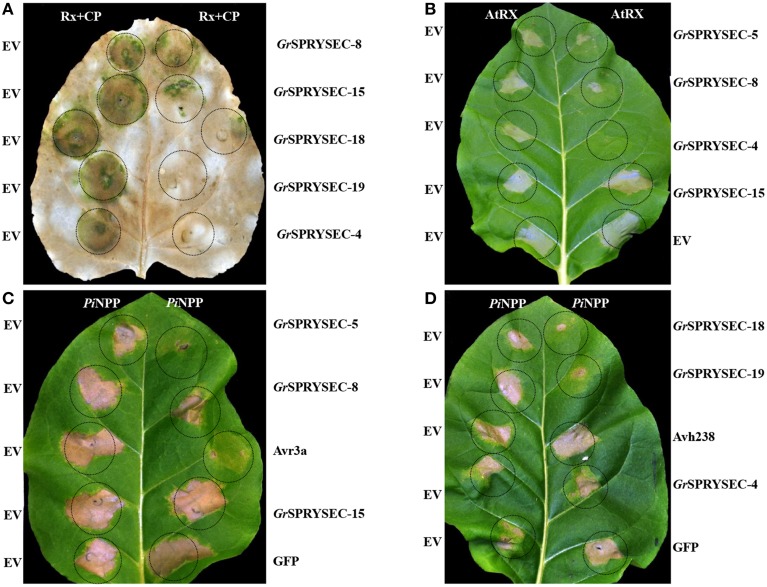
**Suppression of cell death induced by NB-LRR and ***Pi***NPP proteins by ***Gr***SPRYSECs in ***N. benthamiana*** and ***N***. ***tabacum***. (A)** *N. benthamiana* leaves were co-infiltrated with *Agrobacterium* carrying binary vectors expressing Rx, CP and P38 together with either empty vector (EV, left hand side) or the indicated effectors expressed from pEAQ35S (right hand side). Three days DPI, leaves were decolorized with methanol to highlight cell death reactions. **(B)** *N. tabacum* leaves were co-infiltrated with *Agrobacterium* containing binary vectors expressing AtRx and P38 together with either empty vector (EV, left hand side) or the indicated effectors expressed from pEAQ35S (right hand side). **(C,D)** *N. tabacum* leaves were co-infiltrated with *Agrobacterium* carrying expression vectors for *Pi*NPP and P38 together with either empty vector (EV, left hand side) or the indicated effectors (right hand side). Effectors were expressed from a PVX expression vector in **(C,D)**. Cell death symptoms were scored at 3-5 DPI and the pictures were taken at 5 DPI.

Many microbial and metazoan effectors have been reported to suppress defense-related cell death when expressed in plant cells, which likely reflects their virulence function (Postma et al., 2012; Win et al., 2012; Goverse and Smant, 2014; Jaouannet et al., 2014). We tested the SPRYSEC effectors for their ability to suppress cell death by transiently co-agroinfiltrating them in *N. benthamiana* and *N. tabacum* leaves either from binary (Figures 2A,B, Table 1) or PVX expression vectors together with several defense-related cell death inducers, including the Rx protein together with its elicitor, the PVX coat protein (CP), AtRx and the *P. sojae* elicitor PiNPP (Bendahmane et al., 2002; Kanneganti et al., 2006). Cell death inducers were expressed with either SPRYSEC proteins or with empty vector, along with P38, the viral suppressor of RNA silencing of *Turnip crinkle virus* (TCV) (Qu et al., 2003; Thomas et al., 2003) to ensure sustained expression. Avr3a and Avh238 were included as positive and negative controls, respectively. Representative experiments from selected effectors are shown (Figures 2A,B and Figure S2). In *N. benthamiana* the cell death induced by Rx plus CP was completely suppressed by *Gr*SPRYSEC-4, *Gr*SPRYSEC-18, and *Gr*SPRYSEC-19, while suppression of cell death by *Gr*SPRYSEC-5 and *Gr*SPRYSEC-8 was partial (Figure 2A, Table 1). With the exception of *Gr*SPRYSEC-15, which induces an HR on its own (Figure 1A), these effectors also suppressed the cell death induced by AtRx in *N. tabacum* (Figure 2B, Table 1). Likewise, GrSPRYSEC proteins inhibited the cell death induced by an additional NB-LRR protein, Bs2, when expressed with its cognate effector AvrBs2 in *N. tabacum* (Figure S3, Table 1; Tai et al., 1999). When expressed from a PVX vector, the same effectors also completely or partially suppressed the cell death induced by PiNPP in *N. tabacum* (Figures 2C,D) and in *N. benthamiana* (Figure S2). These results suggest that SPRYSEC effectors of *G. rostochiensis* can suppress cell death associated with defense responses induced both by NB-LRR proteins as well as by an elicitor.

**Table 1:** **Suppression of cell death by ***G. rostochiensis*** SPRYSEC proteins**.

	**Suppression of cell death in *N. benthamiana*^b^**		**Suppression of cell death in *N. tabacum*^b^**		
**Effector^a^**	**AtRx**	***Pi*NPP**	**AtRx**	***Pi*NPP**	**Bs2/AvrBs2**
*Gr*SPRYSEC-4	+	++	++	+	++
*Gr*SPRYSEC-5	+	++	++	+	++
*Gr*SPRYSEC-8	+	++	+	++	+
*Gr*SPRYSEC-15	+	++	−	−	−
*Gr*SPRYSEC-18	+	+	+	+	+++
*Gr*SPRYSEC-19	+	++	++	+	++
*P. infestans* Avr3a	Nt	++	Nt	+	Nt
*P. sojae* Avr3b	+	Nt	+	+	+
pGR106-empty	−	−	−	−	−
pGR106-GFP	−	−	−	−	−
pEAQ35S-empty	−	−	−	−	−
pEAQ35S-GFP	−	−	−	−	−

a *Effectors were expressed from pEAQ35S or PVX based constructs, as described in the text*.

b *Cell death suppression was assessed visually and assigned to categories with cell death suppressed in at least 75% of infiltrated patches (+++); in at least 50% of infiltrated patches (++); in at least 25% of infiltrated patches (+); no suppression or less than 25% (−); (Nt) Not tested*.

### Suppression of disease resistance mediated by the NB-LRR proteins Rx and N

**Figure 3:**
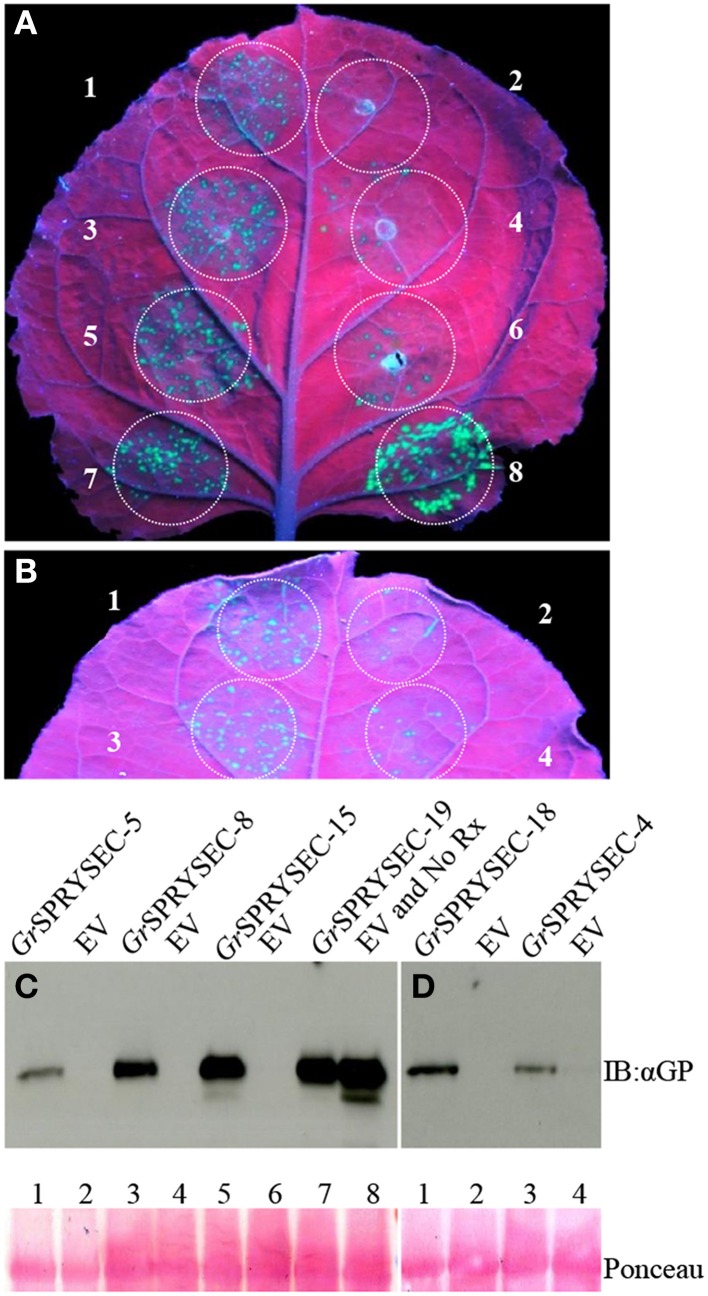
*****Gr***SPRYSECs suppress Rx-mediated resistance to PVX**. *N. benthamiana* leaves were co-infiltrated with *Agrobacterium* carrying binary vectors expressing PVX-GFP and Rx together with **(A)** 1, *Gr*SPRYSEC-5; 2, empty vector; 3, *Gr*SPRYSEC-8; 4, empty vector; 5 *Gr*SPRYSEC-15; 6, empty vector; 7, *Gr*SPRYSEC-19; 8, empty vector; and Rx replaced with empty vector. **(B)** 1, *Gr*SPRYSEC-18; 2, empty vector; 3, *Gr*SPRYSEC-4; 4, empty vector; GFP expression was visualized and photographed under UV illumination at 4 DPI. **(C,D)** Anti GFP immune blotting was performed on total protein samples taken at 4 DPI from infiltrated *N. benthamiana* leaf patches expressing the different construct combinations as described in **(A,B)**. Numbering corresponds to the number on the leaf above each blot. Ponceau staining (lower panel) was used to show equal loading.

**Figure 4:**
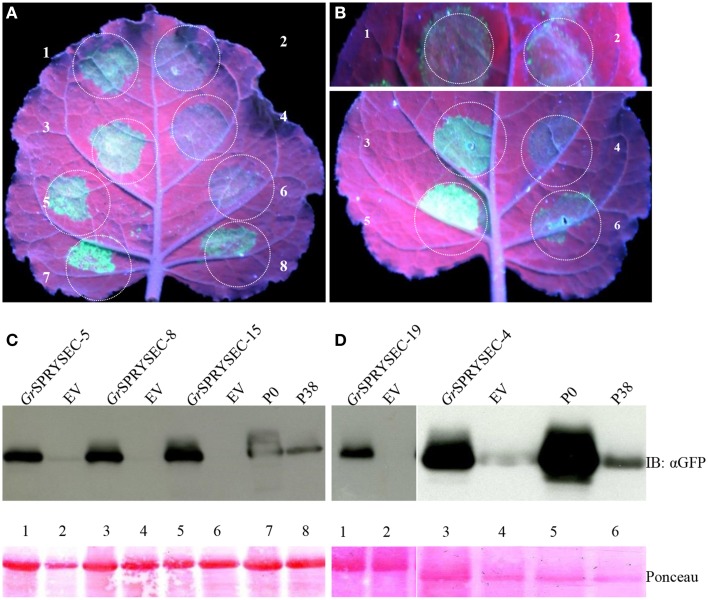
*****Gr***SPRYSECs proteins suppress virus resistance mediated by the ***N gene*****. *N. benthamiana* leaves were co-infiltrated with *Agrobacterium* carrying binary vectors expressing PVX-GFP, N, and P50 together with **(A)** 1, *Gr*SPRYSEC-5; 2, empty vector; 3, *Gr*SPRYSEC-8; 4, empty vector; 5, *Gr*SPRYSEC-15; 6, empty vector; 7, P0; 8, P38. **(B)** 1, *Gr*SPRYSEC-19; 2, empty vector; 3, *Gr*SPRYSEC-4; 4, empty vector; 5, P0; 6, P38. GFP expression was visualized under UV illumination at 4 DPI. **(C,D)** Anti GFP immune blotting was performed on total protein samples taken at 4 DPI from *N. benthamiana* leaf patches co-expressing the combinations of constructs described in **(A,B)**. The number on the blot corresponds to the number on the leaf above each blot. Ponceau staining (lower panel) was used to show equal loading.

Although a number of effectors have been shown to repress defense-related cell death, cell death is not an absolute requirement for halting pathogen proliferation in plants, suggesting that additional mechanisms contribute to immunity, which may or may not be repressed by a given effector. We investigated whether the SPRYSEC proteins could also suppress disease resistance mediated by the Rx and N proteins, which confer resistance to viruses without inducing cell death (Bendahmane et al., 1999; Bhattacharjee et al., 2009). To test this, PVX-GFP was agroinfiltrated in *N. benthamiana* leaves with the *Rx* gene along with either empty vector as a control or the SPRYSEC effectors. GFP was then visualized by UV illumination and immuno-blotting 4 days later as a proxy for virus accumulation in *N. benthamiana* leaves. In the leaf patches co-agroinfiltrated with PVX-GFP, Rx, and empty vector, little, or no GFP was observed, whereas all SPRYSEC effectors allowed significant accumulation of GFP in the infiltrated areas as observed visually and by anti-GFP immune-blotting (Figure 3). As a further demonstration that the SPRYSEC proteins can inhibit defense responses other than cell death, we used an assay based on the *N* gene, which confers resistance to *Tobacco mosaic virus* (TMV) through the recognition of the P50 subunit of the viral replicase (Bhattacharjee et al., 2009). The co-expression of N and P50 in *N. benthamiana* leaves inhibits the accumulation of PVX-GFP in the absence of cell death (Bhattacharjee et al., 2009). We co-expressed N and P50 with PVX-GFP together with either empty vector or the SPRYSEC effectors and monitored the accumulation of PVX-GFP visually and by immuno-blotting. The P0 protein from polerovirus was used as a positive control in this assay as it has been shown to inhibit N-mediated anti-viral defense responses (Bhattacharjee et al., 2009). P0 and all tested SPRYSEC effectors inhibited the ability of N to suppress PVX-GFP accumulation in this assay (Figure 4) indicating that they are able to inhibit the cell death-independent defense pathways induced by N.

## Discussion

To successfully invade plants, phytopathogenic nematodes must degrade or modify host cell walls and reprogram cellular identity and metabolism through the activity of their effectors. At the same time, like all pathogens, nematodes must also inhibit plant defenses. Indeed, we and others have previously shown that *G. rostochiensis* effectors SPRYSEC-19, UBCEP12, SKP1, and EXPB2 can inhibit defense-related cell death and other immune responses (Postma et al., 2012; Chen et al., 2013; Chronis et al., 2013; Ali et al., 2015). However, EXPB2, and possibly SKP1 and UBCEP12, appear to exert their defense repressing activities in the apoplast (Ali et al., 2015). Thus, although UBCEP12 may also function in the cytoplasm (Chronis et al., 2013), intracellular immune suppression by *G. rostochiensis* may to be largely mediated by SPRYSEC proteins. All six of the *G. rostochiensis* SPRYSEC proteins we have characterized possess this ability. In addition, when expressed systemically *in planta*, aside from the induction of certain defense responses (see below), the SPRYSEC proteins did not appear to induce any additional phenotypes in *Nicotiana* spp., in contrast to UBCEP12, SKP1, and EXPB2, which markedly affect plant development (Ali et al., 2015). Thus, although additional SPRYSEC proteins may be present in the *G. rostochiensis* genome (Postma et al., 2012), from our studies we suggest that defense repression is the major function of these proteins.

In agreement with previous reports, we show that *Gr*SPRYSEC-19 (Postma et al., 2012), as well as five additional SPRYSECs were able to inhibit defense responses. SPRYSEC family members appear to be a significant percentage of the intracellular effectors from *Globodera* spp. For example, *G. rostochiensis* possesses 12 or more SPRYSEC proteins whereas the *G. pallida* genome is predicted to encode up to 299 SPRYSEC-containing proteins (Jones et al., 2009; Postma et al., 2012; Cotton et al., 2014). Thus, these proteins appear to constitute one of the largest effector families in plant-parasitic nematodes but appear to be restricted to the genus *Globodera*. The reason for the expansion and diversification of this effector family is not clear, although there may be some functional diversification of these proteins. Some *G. pallida* SPRYSEC proteins have been shown to be localized to the cytoplasm while others seem to be targeted to the nucleus of plant cells (Thorpe et al., 2014). However, the fact that all six SPRYSEC proteins tested here can suppress defenses suggests that defense suppression may be a core property of most or all SPRYSEC proteins in addition to any other, as yet uncharacterized functions of these proteins. Consistent with such a role, SPRYSEC-encoding genes are expressed in the dorsal oesophageal gland cell (Rehman et al., 2009; Thorpe et al., 2014), are upregulated in pre-J2s and their expression is maintained at high levels during early stages of nematode parasitism (Figure S1; Rehman et al., 2009; Thorpe et al., 2014).

All of the *G. rostochiensis* SPRYSEC effectors that suppress HR also abrogate the resistance to PVX-GFP induced either by Rx or N/P50 protein. This assay measures resistance against a virus, but it is likely that the initial signaling pathways that lead to virus resistance are the same as those that lead to resistance to other pathogens upon elicitation of an NB-LRR protein. Since this assay is independent of cell death it suggests that these effectors directly interfere with defense signaling rather than having some generalized role in counteracting cellular stress. Although the mechanism of cell death/defense suppression by *G. rostochiensis* effectors is not clear, it is possible that they interfere with signal transduction at a point where R protein-mediated and PAMP-like elicitor signaling pathways converge. Alternatively, they may target multiple pathways. In a study of *P. sojae* effectors, most of the 169 tested effectors could suppress cell death induced by BAX, INF1 or ETI in *N. benthamiana* (Wang et al., 2011). Similarly in *Hyaloperonospora arabidopsidis* the majority of the candidate effectors tested suppressed host plant immunity (Fabro et al., 2011). In *P. syringae* pv tomato strain DC3000 a majority of its 36 type III effectors have been reported to suppress ETI-associated cell death (Guo et al., 2009). This may indicate a higher degree of redundancy in effectors suppressing cell death in these pathogens compared to *Globodera* spp., which may have expanded the SPRYSEC protein family to allow for similar redundancy. The question of why so many functionally redundant effectors are maintained in pathogen genomes remains, however.

Certain variants of the SPRYSEC protein *Gp*-RBP-1 has been shown to be recognized by the NB-LRR resistant protein Gpa2 (Sacco et al., 2009). This recognition is determined by a single amino acid changes, which appears to be under selective pressure in natural populations (Carpentier et al., 2012), suggesting that this family of protein might be under strong selection pressure to avoid recognition by host resistance proteins. Many effectors elicit defense responses, possibly because, in tampering with the host defense response, they inadvertently set off the response they were meant to defuse (van der Hoorn and Kamoun, 2008; Collier and Moffett, 2009). As such, one could predict that those effectors that suppress defense response might be the most likely to be recognized by the plant innate immune system. Indeed, the apoplastic *Gr*VAP1 protein (which did not suppress cell death in our assay (Ali et al., 2015) as it interferes with a different type of defense) interferes with protease-based defenses and in doing so can elicit a resistance response via the Cf-2 protein (Lozano-Torres et al., 2012). *Gr*SPRYSEC-15 also appears to function as a typical avirulence protein in *N. tabacum*, most likely recognized by an endogenous NB-LRR protein, as it exhibits species specific HR induction and confers resistance to a recombinant virus (Figure 1C). *Gr*SPRYSEC-15 also differs from all other SPRYSECs tested here in that it induced severe stunting and chlorosis in *N. benthamiana* (Figure 1E). This however, is likely to be the result of a weak recognition of this effector by an NB-LRR protein in *N. benthamiana* similar to the effect of AvrB expression in Arabidopsis, which induces chlorosis due to weak activation of the NB-LRR protein TAO1 (Eitas et al., 2008). Why tobacco recognizes *Gr*SPRYSEC-15 is not clear. However, we have shown with other pathogen effectors that it is not unusual for homologs of proteins recognized by the immune system of one plant to be recognized by that of another, non-host plant (Vega-Arreguín et al., 2014; Wang et al., 2015). At the same time, it is possible that the tobacco disease resistance gene that recognizes SPRYSEC-15 may recognize a similar protein from *Globodera* species that infect tobacco, such as the tobacco cyst nematode. At the same time, *Gr*SPRYREC-15 may activate a tobacco NB-LRR protein because it interferes with similar host proteins targeted by the effectors of other tobacco pathogens. Indeed, given that at least two SPRYSEC proteins elicit NB-LRR proteins, we suggest that this class of proteins may have inherent properties, quite possibly related to their ability to inhibit defenses, that predispose them to being recognized by the plant immune system, and that SPRYSEC proteins may function as avirulence determinants in other contexts. As such, future studies aimed at identifying sources of resistance to potato cyst nematodes may focus on searching for recognition of SPRYSEC proteins.

### Conflict of interest statement

The authors declare that the research was conducted in the absence of any commercial or financial relationships that could be construed as a potential conflict of interest.

## Supplementary material

Figure S1 **Expression profile of *Gr*SPRYSEC-encoding genes in different life stages of *Globodera rostochiensis***. The relative expression of six SPRYSEC-encoding genes was determined using quantitative RT-PCR in five *G. rostochiensis* life stages: cyst, pre-parasitic second-stage juvenile (pre-J2) and parasitic second-, third- and fourth-stage juveniles (par-J2, J3, and J4). Values are means ± SD of two biological replicates, normalized to the *G. rostochiensis* β-*actin* gene (*Gract-1*) (EF437156) and relative to expression in the cyst stage.

Figure S2 **Suppression of the HR induced by the *Phytophthora infestans* elicitor *Pi*NPP in *N. benthamiana* by *Gr*SPRYSECs. (A,B)** *N. benthamiana* leaves were co-infiltrated with *Agrobacterium* carrying expression vectors for *Pi*NPP and P38 together with either empty vector (EV, left hand side) or the indicated effectors (right hand side). Effectors were expressed from a PVX expression vector. Cell death symptoms were scored at 3–5 DPI and photographed at 5 DPI.

Figure S3 **Suppression of the HR by *Gr*SPRYSECs in *N. tabacum* induced by AvrBs2 and Bs2. (A,B)** *N. tabacum* leaves were co-infiltrated with *Agrobacterium* carrying expression vectors for AvrBs2/BS2 and P38 together with either empty vector (EV, left hand side) or the indicated effectors (right hand side). Effectors were expressed from a PVX expression vector. Cell death symptoms were scored at 3–5 DPI and photographed at 5 DPI.

Figure S4 **Sequence alignment of *Gr*SPRYSEC-4 and *Gr*SPRYSEC-15 with corresponding reference genes**. Nucleotide sequences of previously described reference genes (ref; see Table S2) vs. those found in the Québec populations (QC) used in this study were aligned. Shown in red are predicted coding sequences, excluding the portion of the mRNA encoding the signal peptide, which was not amplified in this study. **(A)** Sequence of reference *GrSPRYSEC-4* (JX026913.1) compared to *GrSPRYSEC-4* (K963513.1) reported herein. **(B)** Sequence of reference *GrSPRYSEC-15* (JX026918.1) compared to *GrSPRYSEC-15* (K963515.1) reported herein.

Table S1 **Primers used in this work**.

Table S2 **Sequence variation from reference genes of SPRYSEC-coding genes from *Globodera rostochiensis***.
