# Supplementary figures and images for: Analysis of Globodera rostochiensis effectors reveals conserved functions of SPRYSEC proteins in suppressing and eliciting plant immune responses
Source: Front Plant Sci. 2015 Aug 11;6:623. doi: 10.3389/fpls.2015.00623 (PMC4532164; doi:10.3389/fpls.2015.00623)

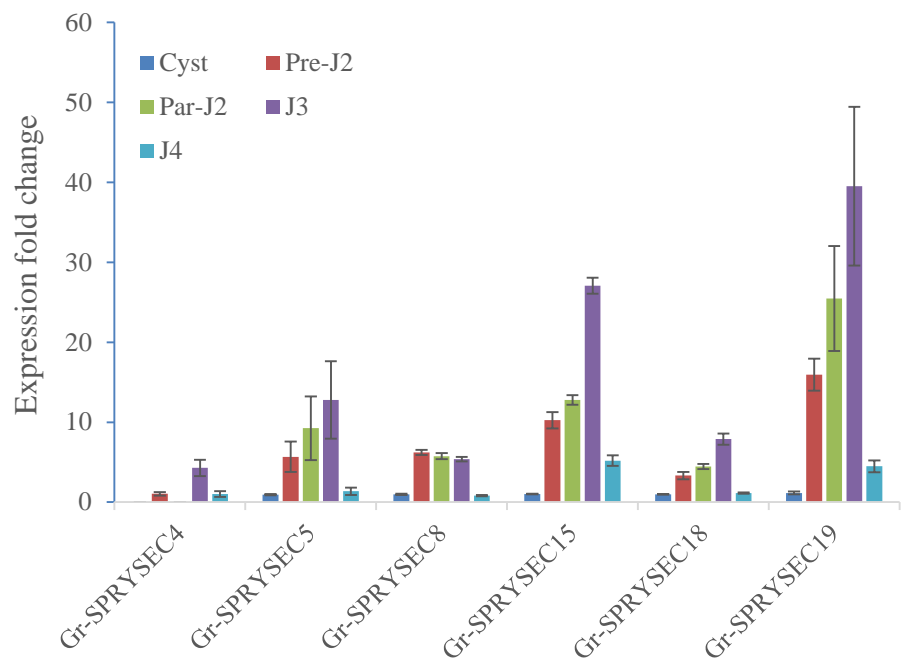

Supplement: Figure S1 — Expression profile of GrSPRYSEC-encoding genes in different life stages of Globodera rostochiensis. The relative expression of six SPRYSEC-encoding genes was determined using quantitative RT-PCR in five G. rostochiensis life stages: cyst, pre-parasitic second-stage juvenile (pre-J2) and parasitic second-, third- and fourth-stage juveniles (par-J2, J3, and J4). Values are means ± SD of two biological replicates, normalized to the G. rostochiensis β-actin gene (Gract-1) (EF437156) and relative to expression in the cyst stage. [file Image1.PDF]

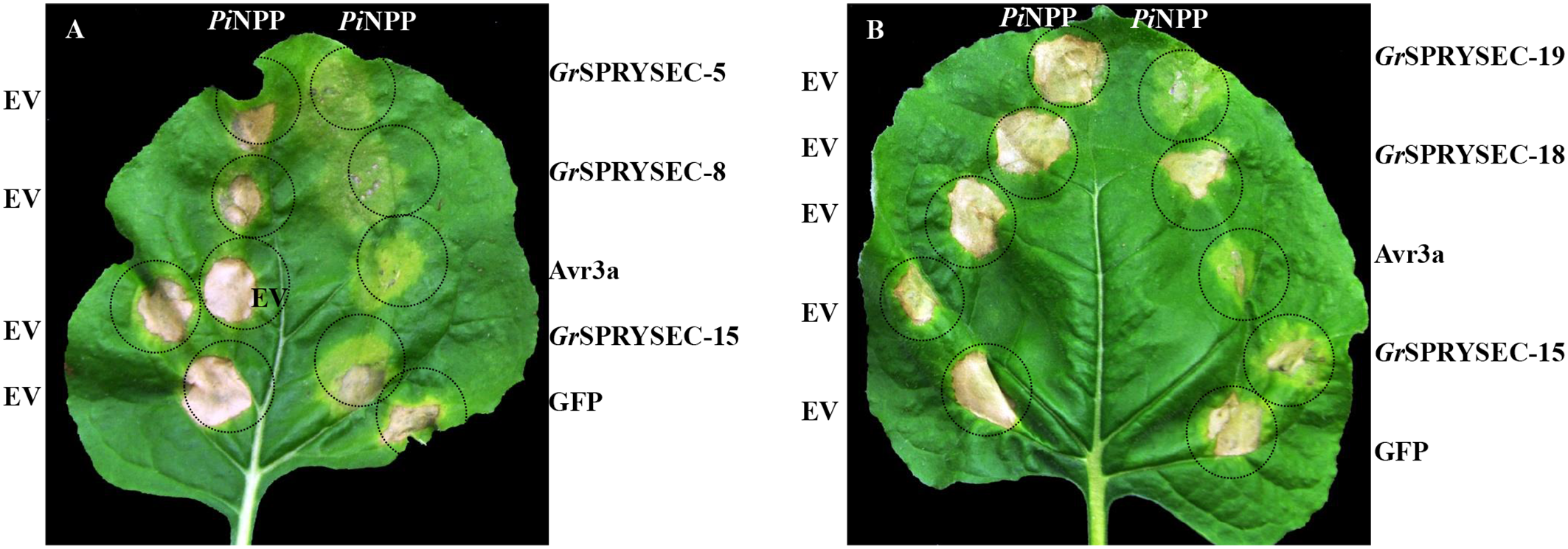

Supplement: Figure S2 — Suppression of the HR induced by the Phytophthora infestans elicitor PiNPP in N. benthamiana by GrSPRYSECs. (A,B) N. benthamiana leaves were co-infiltrated with Agrobacterium carrying expression vectors for PiNPP and P38 together with either empty vector (EV, left hand side) or the indicated effectors (right hand side). Effectors were expressed from a PVX expression vector. Cell death symptoms were scored at 3–5 DPI and photographed at 5 DPI. [file Image2.TIF]

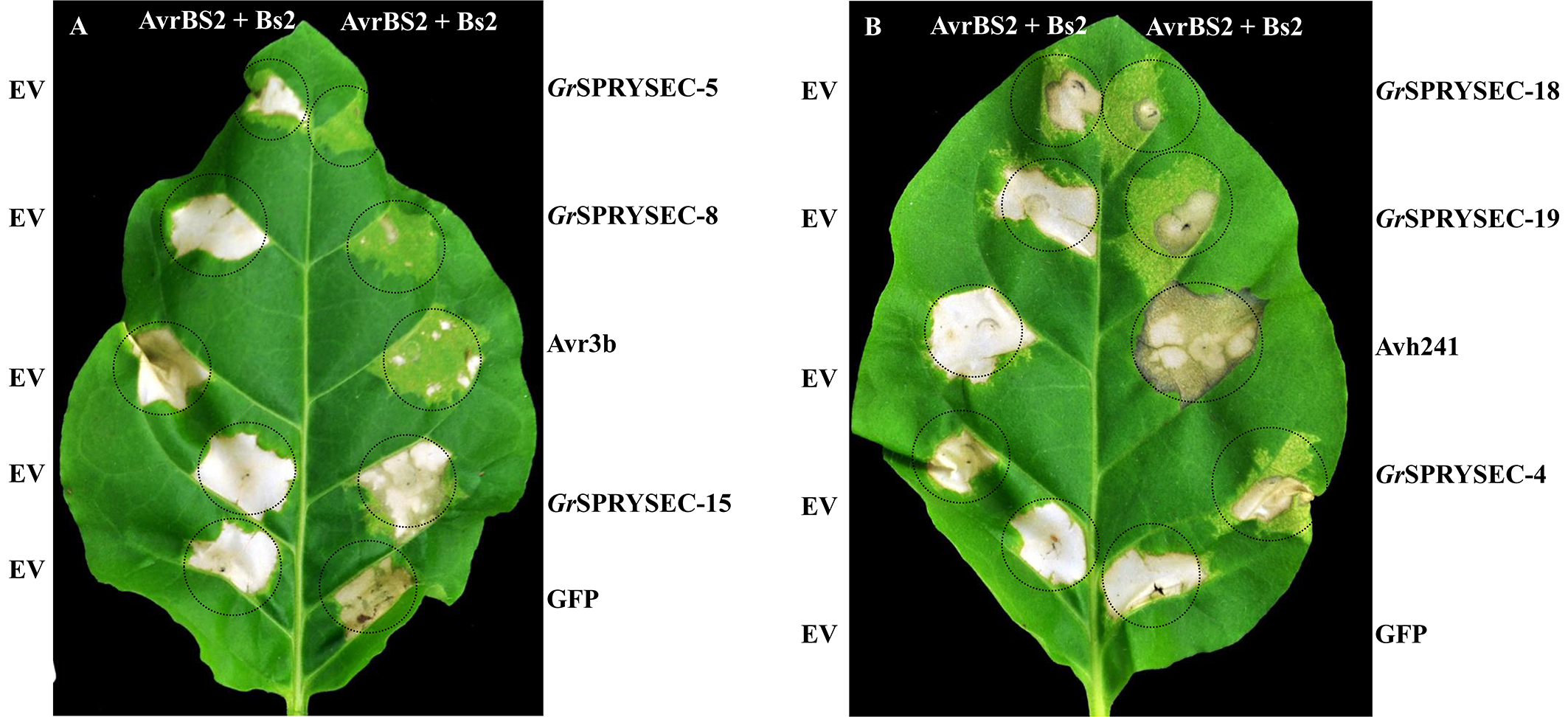

Supplement: Figure S3 — Suppression of the HR by GrSPRYSECs in N. tabacum induced by AvrBs2 and Bs2. (A,B) N. tabacum leaves were co-infiltrated with Agrobacterium carrying expression vectors for AvrBs2/BS2 and P38 together with either empty vector (EV, left hand side) or the indicated effectors (right hand side). Effectors were expressed from a PVX expression vector. Cell death symptoms were scored at 3–5 DPI and photographed at 5 DPI. [file Image3.TIF]
