# Supplementary material for: Analysis of Globodera rostochiensis effectors reveals conserved functions of SPRYSEC proteins in suppressing and eliciting plant immune responses
Source: Front Plant Sci. 2015 Aug 11;6:623. doi: 10.3389/fpls.2015.00623 (PMC4532164; doi:10.3389/fpls.2015.00623)
Supplement: Figure S4 — Sequence alignment of GrSPRYSEC-4 and GrSPRYSEC-15 with corresponding reference genes. Nucleotide sequences of previously described reference genes (ref; see Table S2) vs. those found in the Québec populations (QC) used in this study were aligned. Shown in red are predicted coding sequences, excluding the portion of the mRNA encoding the signal peptide, which was not amplified in this study. (A) Sequence of reference GrSPRYSEC-4 (JX026913.1) compared to GrSPRYSEC-4 (K963513.1) reported herein. (B) Sequence of reference GrSPRYSEC-15 (JX026918.1) compared to GrSPRYSEC-15 (K963515.1) reported herein. [file Image4.PDF]

A

```
                   1        10        20        30        40        50        60        70        80        90        100       110       120       130
GrSPRYSEC-4 (ref)  |--------+---------+---------+---------+---------+---------+---------+---------+---------+---------+---------+---------+---------|
GrSPRYSEC-4 (QC)   ATGCGCACCACCATTCTCCTCTTGGGCGCTGGTTGCTTGATCGTCGCCTCTATTCTGCTGGAGACGGATGCATCGCTGCCAAAAACGTTGCCATCAAACACAAACACAGAGCAAAATCAATGGGATGCCG
Consensus                                                                                  TCGCTGCCAAAAACGTTGCCATCAAACACAAACACAGAGCAAAATCAATGGGATGCCG
                   ........................................................................TCGCTGCCAAAAACGTTGCCATCAAACACAAACACAGAGCAAAATCAATGGGATGCCG

                   131      140       150       160       170       180       190       200       210       220       230       240       250       260
GrSPRYSEC-4 (ref)  |--------+---------+---------+---------+---------+---------+---------+---------+---------+---------+---------+---------+---------|
GrSPRYSEC-4 (QC)   ATACATGTAACAAGGGCCTCACGTTGTCTGTCTCCACACTCTCTGAGCCAGAACAATTGCCTGGCTCCAGATTGATTGTCAAATATAATGAATATAATTACGAGTCGCGCTCTATCCGCGCTGAACAGCC
Consensus          ATACATGTAACAAGGGCCTCACGTTGTCTGTCTCCACACTCTCTGAGCCAGAACAATTGCCTGGCTCCAGATTGATTGTCAAATATAATGAATATAATTACGAGTCGCGCTCTATCCGCGCTGAACAGCC
                   ATACATGTAACAAGGGCCTCACGTTGTCTGTCTCCACACTCTCTGAGCCAGAACAATTGCCTGGCTCCAGATTGATTGTCAAATATAATGAATATAATTACGAGTCGCGCTCTATCCGCGCTGAACAGCC

                   261      270       280       290       300       310       320       330       340       350       360       370       380       390
GrSPRYSEC-4 (ref)  |--------+---------+---------+---------+---------+---------+---------+---------+---------+---------+---------+---------+---------|
GrSPRYSEC-4 (QC)   AATTACAAAAAGTAAATCCGACATGTTCTACTACGAAGTGAAAATATTAGCGTTAGCGCTAACAGGTATTATTTCCATTGGAGTTGGTCCCAAACAAATGCCATTGAACAAAGAGATTGGAATGCTCAAA
Consensus          AATTACAAAAAGTAAATCCGACATGTTCTACTACGAAGTGAAAATATTAGCGTTAGCGCTAACAGGTATTATTTCCATTGGAGTTGGTCCCAAACAAATGCCATTGAACAAAGAGATTGGAATGCTCAAA
                   AATTACAAAAAGTAAATCCGACATGTTCTACTACGAAGTGAAAATATTAGCGTTAGCGCTAACAGGTATTATTTCCATTGGAGTTGGTCCCAAACAAATGCCATTGAACAAAGAGATTGGAATGCTCAAA

                   391      400       410       420       430       440       450       460       470       480       490       500       510       520
GrSPRYSEC-4 (ref)  |--------+---------+---------+---------+---------+---------+---------+---------+---------+---------+---------+---------+---------|
GrSPRYSEC-4 (QC)   GGCACTTACGCATACGGAAGCGGCCGTAAGGCCTTTATTTGGGGTCACGAGCTCGCGGGATGTCGGCACGCCAATGGGCGTCCTTACATCTTCGAAGGAATTCCCGAGTTTCGTGCCGATGACGTGATCG
Consensus          GGCACTTACGCATACGGAAGCGGCCGTAAGGCCTTTATTTGGGGTCACGAGCTCGCGGGATGTCGGCACGCCAATGGGCGTCCTTACATCTTCGAAGGAATTCCCGAGTTTCGTGCCGATGACGTGATCG
                   GGCACTTACGCATACGGAAGCGGCCGTAAGGCCTTTATTTGGGGTCACGAGCTCGCGGGATGTCGGCACGCCAATGGGCGTCCTTACATCTTCGAAGGAATTCCCGAGTTTCGTGCCGATGACGTGATCG

                   521      530       540       550       560       570       580       590       600       610       620       630       640       650
GrSPRYSEC-4 (ref)  |--------+---------+---------+---------+---------+---------+---------+---------+---------+---------+---------+---------+---------|
GrSPRYSEC-4 (QC)   GATGCGGTGTCAATTTGAAAAATCGCAAAATCTTTTACACAAAGAATGGAGTGCTTTTAGAAACGCCCGTTTTGTTAGTCGATTCTGTCGCCGGATTGTTCCCATGGGTTACGTTGTATTGGATAGGAGA
Consensus          GATGCGGTGTCAATTTGAAAAATCGGAAAATCTTTTACACAAAGAACGGAGTGCTTTTAGAAACGCCCGTTTTGTTAGTCGATTCTGTCGCCGGATTGTTCCCATGGGTTACGTTGTATTGGATAGGAGA
                   GATGCGGTGTCAATTTGAAAAATCGcAAAATCTTTTACACAAAGAAcGGAGTGCTTTTAGAAACGCCCGTTTTGTTAGTCGATTCTGTCGCCGGATTGTTCCCATGGGTTACGTTGTATTGGATAGGAGA

                   651      660       670       680       690      699
GrSPRYSEC-4 (ref)  |--------+---------+---------+---------+--------|
GrSPRYSEC-4 (QC)   CGCAATTAAAGCAAACTTTGGACCGAACTTTGTGTACAAATTCGCATGA
Consensus          CGCAATTAAAGCAAACTTTGGACCGAACTTTGTGTACAAATTCGCATGA
                   CGCAATTAAAGCAAACTTTGGACCGAACTTTGTGTACAAATTCGCATGA
```

B

```
                    1        10        20        30        40        50        60        70        80        90        100       110       120       130
                    |--------+---------+---------+---------+---------+---------+---------+---------+---------+---------+---------+---------+---------|
GrSPRYSEC-15 (ref)  ATGCCCACCTTTCTCTTCTTGGCCGCAATTTGTTTGCTTGTGGCCGCTTCAATTCTGCTGCAAACTGATGCTTCGCCAAAAGCAAACGCCAAACTCGAAAAAGTACCATCTGGCAACGCTGAATCAACCC
GrSPRYSEC-15 (QC)   ..................................................................................................................................
       Consensus

                    131      140       150       160       170       180       190       200       210       220       230       240       250       260
                    |--------+---------+---------+---------+---------+---------+---------+---------+---------+---------+---------+---------+---------|
GrSPRYSEC-15 (ref)              TTCAAAATCGATGGGCATATTCCGCACGTGACGAGAAACTCGCGTGGGATTCCGCTGCACGTGACGAGAAACTCGAGCTCACTGAGCCCGCCGGATTGATTGTTCAATTTATTGGAGA
  SPRYSEC-15 (QC)  CAGTGCTAACCCTTCAAAATCGATGGGCATATTCCGCACGTGACGAGAAACTCGCGTGGGATTCCGCTGCACGTGACGAGAAACTCGAGCTCACTGAGCCCGCCGGATTGATTGTTCAATTTATTGGAGA
       Consensus    ............TTCAAAATCGATGGGCATATTCCGCACGTGACGAGAAACTCGCGTGGGATTCCGCTGCACGTGACGAGAAACTCGAGCTCACTGAGCCCGCCGGATTGATTGTTCAATTTATTGGAGA

                    261      270       280       290       300       310       320       330       340       350       360       370       380       390
                    |--------+---------+---------+---------+---------+---------+---------+---------+---------+---------+---------+---------+---------|
GrSPRYSEC-15 (ref)  GAATAGCAAGCATCGCTCTGTCCGCGCTAAATTGCCAATTCCAAAAGGGAATTCTGGCATTTTTTACTACGAAGTGACCATCTCGGGGGATGGAGACGCTATTTACATTGGACTTGCAACAGAACAAATG
GrSPRYSEC-15 (QC)   GAATAGCAAGCATCGCTCTGTCCGCGCTAAATTGCCAATTCCAAAAGGGAATTCTGGCATTTTTTACTACGAAGTGACCATCTCGGGGGATGGAGACGCTATTTACATTGGACTTGCAACAGAACAAATG
       Consensus    GAATAGCAAGCATCGCTCTGTCCGCGCTAAATTGCCAATTCCAAAAGGGAATTCTGGCATTTTTTACTACGAAGTGACCATCTCGGGGGATGGAGACGCTATTTACATTGGACTTGCAACAGAACAAATG

                    391      400       410       420       430       440       450       460       470       480       490       500       510       520
                    |--------+---------+---------+---------+---------+---------+---------+---------+---------+---------+---------+---------+---------|
GrSPRYSEC-15 (ref)  CCATTGCGGGACACACATGTTGGATATAATGAAGGCACTTACGGATACGGAAGCTCGGGCAAATTTTGGGGTCACGAAGTTGGGGGATGTTCCCACTGGGGTAATGAACGTCCGTACATCGATGGACAGC
GrSPRYSEC-15 (QC)   CCATTGCGGGACACACATGTTGGATATAATGAAGGCACTTACGGATACGGAAGCTCGGGCAAATTTTGGGGTCACGAAGTTGGGGGATGTTCCCACTGGGGTAATGAACGTCCGTACATCGATGGACAGC
       Consensus    CCATTGCGGGACACACATGTTGGATATAATGAAGGCACTTACGGATACGGAAGCTCGGGCAAATTTTGGGGTCACGAAGTTGGGGGATGTTCCCACTGGGGTAATGAACGTCCGTACATCGATGGACAGC

                    521      530       540       550       560       570       580       590       600       610       620       630       640       650
                    |--------+---------+---------+---------+---------+---------+---------+---------+---------+---------+---------+---------+---------|
GrSPRYSEC-15 (ref)  CCAAATTTGACCGTAACAACATCATCGGATGCGGCGTGAATTTGAAAACGCGCCAAATCATTTACACACACAATGGGCGGCCTTTGGAAACTACCGGGCTGTTAGTCGCCGAGTCTGCCGCCGAATTGTA
GrSPRYSEC-15 (QC)   CCAAATTTGACCGTAACAACATCATCGGATGCGGCGTGAATTTGAAAACGCGCCAAATCATTTACACACACAATGGGCGGCCTTTGGAAACTACCGGGCTGTTAGTCGCCGAGTCTGCCGCCGAATTGTA
       Consensus    CCAAATTTGACCGTAACAACATCATCGGATGCGGCGTGAATTTGAAAACGCGCCAAATCATTTACACACACAATGGGCGGCCTTTGGAAACTACCGGGCTGTTAGTCGCCGAGTCTGCCGCCGAATTGTA

                    651      660       670       680       690       700       710       720       730       740       750       760       770       780
                    |--------+---------+---------+---------+---------+---------+---------+---------+---------+---------+---------+---------+---------|
GrSPRYSEC-15 (ref)  CCCGTGCGTTTCGTTGTTCACTTCTGGCAACGAAATTGAAGCGAATTTTGGCACGAAACCATTCAAATT-CAACATTGCCGAAGGCATTTAG
GrSPRYSEC-15 (QC)   CCCGTGCGTTTCGTTGTTCACTTCTGGCAACGAAATTGAAGCGAATTTTGGCACGAAACCATTCAAATTTCAACATTGCCGAAGGCATTTAGAAAATGAAAAAAAAACGAGGGGAAATTATGGGGATTTT
       Consensus    CCCGTGCGTTTCGTTGTTCACTTCTGGCAACGAAATTGAAGCGAATTTTGGCACGAAACCATTCAAATT.CAACATTGCCGAAGGCATTTAG......................................

                    781      790       800       810       820  825
                    |--------+---------+---------+---------+----|
GrSPRYSEC-15 (ref)
GrSPRYSEC-15 (QC)   GTTGTTGAGCGGTTTTCCGTCTTTGTTTTTTTTTCNTTGTGTTGA
       Consensus    .............................................
```
