# Supplementary material for: Analysis of Globodera rostochiensis effectors reveals conserved functions of SPRYSEC proteins in suppressing and eliciting plant immune responses
Source: Front Plant Sci. 2015 Aug 11;6:623. doi: 10.3389/fpls.2015.00623 (PMC4532164; doi:10.3389/fpls.2015.00623)
Supplement: Table S1 — Primers used in this work. [file Table1.DOCX]

| Primer Name | Sequence (5' > 3') |
| --- | --- |
| attB1 For SPRYSEC-15ΔSP | GGGGACAAGTTTGTACAAAAAAGCAGGCTTC AGAACCATGTCGCCAAAAGCAAACG |
| attB1 For SPRYSEC-18ΔSP | GGGGACAAGTTTGTACAAAAAAGCAGGCTTC AGAACCATGCTGGAAACGGATGCATCG |
| attB2 rev SPRYSEC-15 | GGGGACCACTTTGTACAAGAAAGCTGGGTC TCAACACAACGAAAAAAAAACAAAGACG |
| attB2 rev SPRYSEC-18 | GGGGACCACTTTGTACAAGAAAGCTGGGTC TCACTTTCTAAATGCCTCGGCAATGTCG |
| attB1 For SPRYSEC-8ΔSP | GGGGACAAGTTTGTACAAAAAAGCAGGCTTC AGAACCATGTCGCCGGCAGTAGACACAACC |
| attB2 rev SPRYSEC-8 | GGGGACCACTTTGTACAAGAAAGCTGGGTC CTATTTCACATTTTTAATGTTG |
| attB1 For SPRYSEC- 5ΔSP | GGGGACAAGTTTGTACAAAAAAGCAGGCTTC AGAACCATGTCGCCGCCAGAAACATCAAACG |
| attB2 rev SPRYSEC-5 | GGGGACCACTTTGTACAAGAAAGCTGGGTC CTAGTTTTTCAGTTTCAAG |
| attB1 For SPRYSEC-4 ΔSP | GGGGACAAGTTTGTACAAAAAAGCAGGCTTC AGAACCATGTCGCTGCCAAAAACGTTGCCATC |
| attB2 rev SPRYSEC-4 | GGGGACCACTTTGTACAAGAAAGCTGGGTC TCATGCGAATTTGTACACAAAGTTCG |
| SPRYSEC-15-For-207bp | TGCGTTTCGTTGTTCACTTC |
| SPRYSEC-15-For-273bp | GCGCCAAATCATTTACACAC |
| SPRYSEC-5_583F | GAGAAATTTTAATCGCCGGTTT |
| SPRYSEC-5_744R | CAGTTTCAAGTTTTTCAGTTTCAAG |
| SPRYSEC-8_405F | TGAATGGTTTGATGAAAACGAC |
| SPRYSEC-8_574R | CAATTTTGGAGCCTGTGTGATA |
| SPRYSEC-18_302F | CTCGGAGAAAGTAGCCGGTAT |
| SPRYSEC-18_467R | TTAGGGTCAAATTTGGGTTTTC |
| SPRYSEC-19_147F | GATACCGATGCACATCACAAG |
| SPRYSEC-19_415R | AATCCTTGTGACCCCAAAAATA |
| SPRYSEC-15_130F | CCAGTGCTAACCCTTCAAAATC |
| SPRYSEC-15_372R | TCCAATGTAAATAGCGTCTCCA |

**Supplemental Table 1.** Primer sets used for cloning and qRT-PCR of *G. rostochiensis* effectors
